# Supplementary material for: Active Immunization Using TRPM2 Peptide Vaccine Attenuates Atherosclerotic Progression in a Mouse Model of Atherosclerosis
Source: Vaccines (Basel). 2025 Feb 26;13(3):241. doi: 10.3390/vaccines13030241 (PMC11946763; doi:10.3390/vaccines13030241)
Supplement: Supplementary file 1 [file vaccines-13-00241-s001.zip › vaccines-3439700-supplementary.pdf]

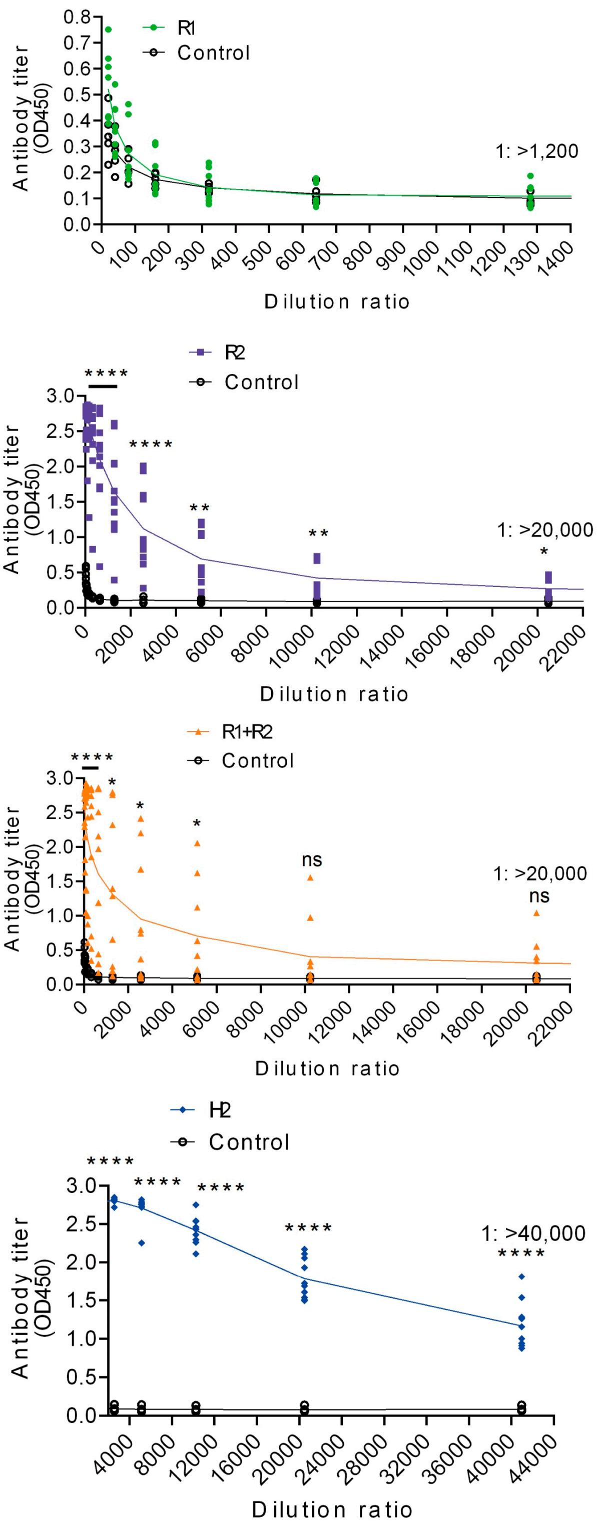

Supplemental Figure S1. Titration of polyclonal antibodies produced in mice in response to different E2 region-based peptide vaccines. Titration of TRPM2 E3-based polyclonal antibody produced in mice in response to different peptide vaccination. The mice were immunized with R1, R2, R1+R2, H2, or KLH control. The polyclonal antiserum was taken from the mice, followed by Elisa-based titration vs. respective synthetic peptides. Shown are titration curve of the antiserum at different dilution. Mean ± SE (n = 5-10). ns = not significant; *p<0.05; **p<0.01; ****, P<0.0001.

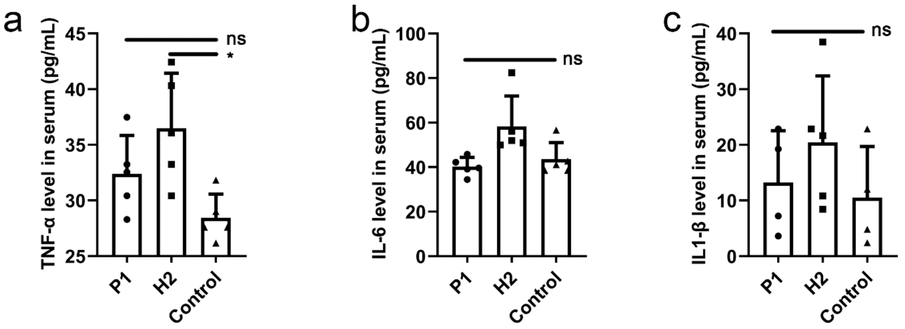

Supplemental Figure S2: The effect of E3 region-based peptide vaccines P1 and H2 on serum levels of inflammatory cytokines TNF-α (a), IL-6 (b) and IL1-β (c). ApoE−/− mice were fed with high cholesterol diet and immunized with or without peptide vaccines. The serum levels of inflammatory cytokines were analysed by ELISA. Data are shown as Mean ± SEM (n = 4-5), * $p < 0.05$; ns, not significant.
